# PDGFA in Cashmere Goat: A Motivation for the Hair Follicle Stem Cells to Activate

**DOI:** 10.3390/ani9020038

**Published:** 2019-01-28

**Authors:** Irene Pazzaglia, Francesca Mercati, Marco Antonini, Stefano Capomaccio, Katia Cappelli, Cecilia Dall’Aglio, Antonietta La Terza, Matteo Mozzicafreddo, Cristina Nocelli, Stefano Pallotti, Dario Pediconi, Carlo Renieri

**Affiliations:** 1 School of Bioscience and Veterinary Medicine, University of Camerino, Via Pontoni 5, 62032 Camerino, Italy; pazzaglia.irene@gmail.com (I.P.); antonietta.laterza@unicam.it (A.L.T.); matteo.mozzicafreddo@unicam.it (M.M.); cristina.nocelli@unicam.it (C.N.); stefano.pallotti@unicam.it (S.P.); dario.pediconi@unicam.it (D.P.); 2 Department of Veterinary Medicine, University of Perugia, Via San Costanzo 4, 06126 Perugia, Italy; stefano.capomaccio@unipg.it (S.C.); katia.cappelli@unipg.it (K.C.); cecilia.dallaglio@unipg.it (C.D.); 3 Italian National Agency for New Technology, Energy and Sustainable Economic Development, ENEA CR Casaccia—SSPT BIOAG Probio, S.M. di Galeria, 00123 Roma, Italy; marco.antonini@enea.it; 4 School of Pharmacy, University of Camerino, Via Gentile III da Varano, 62032 Camerino, Italy; carlo.renieri@unicam.it

**Keywords:** photoperiod, fiber, BMP2, LHX2, PDGFRα, follicular cycle, immunohistochemistry, qPCR

## Abstract

**Simple Summary:**

Cashmere goats are the most important goat breed due to the high yield and fineness of the fibers that they produce. Cashmere fiber is a luxury product since it is soft, light and warm. The development of this fiber depends on the hair follicle (HF) cyclical activity, which is characterized by the succession of growth and regressive phases. In the transition between telogen and anagen phases, many growth factors work to activate the HF stem cells and to allow the growth of a new cashmere fiber. As several factors involved in the stem cell activation, Platelet-Derived Growth Factor A (PDGFA), Bone Morphogenetic Protein 2 (BMP2) and Lim-Homeobox gene 2 (LHX2) were analyzed in this work to evaluate their activity during the cashmere HF cycle. These molecules were studied using different approaches and finally, PDGFA and BMP2 appeared to have higher levels of expression during the cycle activation phase with respect to the LHX2, which suggests that they play a main role in the development of a new cashmere fiber. The obtained data will improve the knowledge of the HF cycle in the cashmere goat and they could be a useful tool for improving cashmere fiber production.

**Abstract:**

The cashmere hair follicle (HF) perpetually goes through cycles of growth, involution and rest. The photoperiod is the main factor in the control of seasonal coat change in cashmere goats while stem cells play a crucial role in the HF growth. Several factors, including Platelet-Derived Growth Factor A (PDGFA), Bone Morphogenetic Protein 2 (BMP2) and Lim-Homeobox gene 2 (LHX2) are implicated in HF morphogenesis and cycle. In this work, the mentioned molecules were investigated to evaluate their role in follicular cycle activation. The study was performed on skin samples collected at different periods of HF cycle and the molecular expression of PDGFA, BMP2 and LHX2 was evaluated by Real-Time PCR (qPCR) at each time point. Since PDGFA showed the most variation, the goat PDGFA gene was sequenced and the protein localization was investigated by immunohistochemistry together with PDGF receptor α (PDGFRα). PDGFA immunostaining was observed in the basal layer of the HF outer root sheath and the immunoreaction appeared stronger in the regressive HFs compared to those in the anagen phase according to qPCR analysis. PDGFRα was observed in the HF epithelium, proving the effect of PDGFA on the follicular structure. The data obtained suggest that PDGFA and BMP2 are both implicated in HF cycle in goat. In particular, PDGFA secreted by the HF is involved in the anagen activation.

## 1. Introduction

Goats are one of the most adaptive livestock species in the world [1] and cashmere goats are the most important goat breed due to the high yield and fineness of the fibers that they produce [2]. China is by far the largest producer of cashmere [3]. The histologic skin structure of the cashmere goat shows two different types of hair follicle (HF): a primary HF that produces guard hair and a secondary HF that gives rise to cashmere hair [4]. Cashmere fiber is soft, light and warm. Its growth starts from the fetal period and continues during the first months after birth. The HF cycle perpetually goes through three stages: growth (anagen), involution (catagen) and rest (telogen) [5]. The photoperiod is the main proximate factor in the control of seasonal coat change. The mitotic activity of secondary HF remains high from the summer to winter solstice before decreasing [6]. The generation of the new hair shaft depends on the activation of hair-specific epithelial stem cells, which are located in the bulge region of the HF placed in the permanent portion of this structure that acts as a reservoir for follicular and sebaceous gland cells [7,8]. In addition, adipocyte precursor cells located in the dermis secrete several growth factors, such as those belonging to the Platelet-Derived Growth Factor (PDGF) family [9], that may be involved in the control and activation of HF [10,11]. PDGF is a potent mitogen produced in a variety of cell types and is important for cell growth, proliferation and differentiation [12,13]. Furthermore, it is active during embryonic development and is found in several adult tissues, including gonads, lung, kidney, intestine, brain and skin [14]. Members of this family are dimeric glycoproteins composed of four different polypeptide chains (A, B, C and D) that are encoded by four different genes [15]. Five isoforms have been described for the PDGF so far: PDGF-AA, PDGF-AB, PDGF-BB, PDGF-CC and PDGF-DD [16]. PDGF signaling was suggested to be instrumental in HF regeneration during the hair cycle [14]. One study suggested that in mouse HF, PDGF induces and maintains the anagen phase [17]. Festa et al. [11] reported that the adipocyte precursor cells secrete PDGF to promote hair growth.

In addition to PDGF, other genes, including Bone Morphogenetic Protein 2 (BMP2) and LIM-Homeobox gene 2 (LHX2), have been assessed for their role in the cashmere cycle since they are involved in HF activation [18,19]. BMP2 is implicated in the regulation of morphogenesis and hair growth. Lee et al. [20] described that BMP2 starts to accumulate from the late anagen phase with a peak at the telogen phase. Others argued that BMP2 is the major molecular driver of bulge quiescence since its expression was absent in the early anagen phase and gradually intensified to reach a peak level in anagen V–VI [21,22].

LHX2 is an important regulator of HF cycle, which controls the switch between stem cell maintenance and activation in the HF [23]. Many authors connected this gene with the beginning of the new hair cycle [24]. Geng et al. [18] found that this gene was active during the HF growth phase and subsided during the resting phase. They compared the LHX2 expression level during the anagen, catagen and telogen phases of the HF cycle and showed that LHX2 is high during the anagen phase and decreases in the other phases [25]. Wang et al. (2016) confirmed that LHX2 regulates the generation and regeneration of the hair in goats.

The aim of the present work was to analyze the expression of PDGFA, BMP2 and LHX2 gene in cashmere goats and determine their variation throughout HF cycle. Since PDGFA showed the greatest difference, the goat PDGFA gene was sequenced and the protein localization was investigated by immunohistochemistry together with the localization of PDGF receptor α (PDGFRα).

## 2. Materials and Methods

### 2.1. Subjects Recruiting and Tissue Collection

The trial was carried out in seven unrelated one-year-old female cashmere goats from the Chianti Cashmere Farm in Radda in Chianti, Italy. Skin samples were collected by a punch biopsy (diameter of 0.8 cm) from the lateral thoracic region after a visual examination to ensure that cutaneous lesions were absent. Biopsies were carried out after trichotomy was performed by using disposable stainless-steel blades, the affected area was carefully washed with povidone-iodine solution and local anesthesia was performed with a subcutaneous injection of 2% lidocaine around the area to be sampled. After the biopsy, the skin lesion was medicated with OneVET^®^ spray (Endospin, La Massimina-Casal Lumbroso, RM, Italy). Samples were collected during four time points at the same time of the day. Collection time and follicular phases were: June (early anagen), September (anagen), December (early catagen) and February (catagen). Each biopsy was divided into two parts: one-half was intended for molecular biology investigations and the other half for morphological evaluations. Biopsies for molecular biology investigations were stored in Allprotect Tissue Reagent (Qiagen GmBH, Hilden, Germany), which preserves the in vivo profile of DNA, RNA and proteins. After this, samples were removed from this solution and put into cryovials (Thermo-Fisher Scientific, Waltham, MA, USA) for storage at −196 °C (liquid nitrogen). Specimens for morphological evaluation were quickly fixed in a 10% formaldehyde solution in phosphate buffered saline (PBS) (0.1 M, pH 7.4) and processed until they were embedded in paraffin wax.

### 2.2. RNA and DNA Isolation

Total RNA was extracted from skin biopsies using the RNeasy Fibrous Tissue Mini Kit (Qiagen GmBH, Hilden, Germany). The quality and quantity of RNA extract was measured using NanoDrop spectrophotometer (Thermo-Fisher Scientific, Waltham, MA, USA) by calculating the optical density ratio at 260/280. The first strand cDNA was synthetized from RNA using SuperScript IV VILO Master Mix (Thermo-Fisher Scientific) according to the manufacturer’s recommendations.

### 2.3. Primer Design

Sequences of PDGFA gene from mammals were chosen from NCBI GenBank (https://www.ncbi.nlm.nih.gov/) and aligned with MAFFT (http://mafft.cbrc.jp/alignment/server/) to determine the conserved regions that were useful for designing primers to amplify this gene. The primers were designed with Primer3 (http://primer3.ut.ee/) (Table 1).

### 2.4. PCR Amplification of Full-Length PDGFA Gene

PCR amplification of the coding region from cDNA was done using the primers FWDA and OligoDT in a reaction volume of 25 µL containing 5X Phusion CG Buffer (Thermo-Fisher Scientific, Waltham, MA, USA), 2 mM dNTP, template, water and 2 U/µL Phusion DNA Polymerase (Thermo-Fisher Scientific) using MJ Mini™ Thermal Cycler (Bio-Rad, Hercules, CA, USA). The PCR cycle was conducted under the following conditions: initial denaturation at 98 °C for 3 min, followed by 35 cycles of 98 °C for 10 s, 63 °C for 20 s and the final extension at 72 °C for 1 min. To obtain the complete sequence of the coding region and the 3′UTR, another PCR was performed using FWDB and OligoDT under these conditions: 98 °C for 3 min, followed by 35 cycles of 98 °C for 10 s, 61 °C for 20 s and the final extension at 72 °C for 50 s. To obtain the 5′ UTR the GeneRacer™ Kit with SuperScript III RT and TOPO TA Cloning (Invitrogen Corp., Carlsbad, CA, USA) was used according to the manufacturer’s recommendations. The PCR reactions were loaded on agarose gel and the fragments of interest were cut and purified using PCR clean-up gel extraction (Macherey-Nagel Inc., Bethlehem, PA, USA). The amplicons were cloned using CloneJET PCR Cloning Kit (Thermo-Fisher Scientific). The sequencing of the transcripts was carried out by StarSEQ (https://www.starseq.com/) before the sequences were determined using NCBI BLAST (https://blast.ncbi.nlm.nih.gov/Blast.cgi).

### 2.5. Homology Modelling of PDGFA Three-Dimensional Structure

The three-dimensional structure of goat PDGFA has not yet been experimentally determined. For this reason, we tried to model its structure by homology in order to determine any significant structural differences. The human PDGFA crystallographic structure [26] was selected as the best template (with a sequence identity of 88.8% and a coverage of 91%) and the Modeller algorithm was used for the modeling [27].

### 2.6. Basic Histology

Sections with a thickness of 5 μm were cut from samples embedded in paraffin wax, mounted onto poly-l-lysine coated glass slides and processed for staining with Hematoxylin & Eosin, Sacpic [28] and Floxin B/Orange G/Alcian blue [29]. Sacpic staining and Floxin B/Orange G/Alcian blue were used to highlight the keratin of the hair and to determine the stage of the HF cycle.

### 2.7. Immunohistochemistry

Immunohistochemistry has been conducted as follows [30]. The skin sections were rehydrated, dipped for 10 min in 3% H_2_O_2_ to reduce endogenous peroxidase activity and incubated for 30 min in 1:10 normal serum (Vector Laboratories, Burlingame, CA, USA). Incubation with 1:400 mouse monoclonal anti-PDGFA and 1:100 rabbit polyclonal anti-PDGFRα antibodies (both from Santa Cruz Biotechnology, Santa Cruz, CA, USA) was performed overnight at room temperature. On the second day, the sections were incubated for 30 min with 1:200 biotin-conjugated secondary antibodies (Santa Cruz Biotechnology), which were namely chicken anti-mouse and chicken anti-rabbit antibodies. The bound primary antibodies were visualized using an avidin-biotin system (Vectastain ABC kit; Vector Laboratories, Burlingame, CA, USA) and diaminobenzidine (DAB) as chromogen (Vector Laboratories). Nuclei were counterstained with Mayer’s Hematoxylin. All steps were performed at room temperature and the slides were incubated in a humid chamber. Sections were washed with PBS between all incubation steps, except after normal serum. Smooth muscle cells were used as an internal positive control [31]. Negative controls were made by omitting the primary antibodies. All sections were observed under a photomicroscope (Nikon Eclipse E800, Nikon Corp., Tokyo, Japan) connected to a digital camera (Nikon Dxm 1200 digital camera, Nikon Corp.).

### 2.8. qPCR on Target Genes

To evaluate the expression of PDGFA, BMP2 and LHX2, a Real-Time PCR (qPCR) approach was applied. For each gene, specific primers spanning exons/introns were designed with Primer-Blast (https://www.ncbi.nlm.nih.gov/tools/primer-blast/) (Table 2).

Three reference genes were chosen according to reference [32], which selected and validated eight reference genes in the skin of Liaoning cashmere goats. The Real-Time PCR was performed using SsoAdvanced Universal SYBR Green Supermix (Bio-Rad, Hercules, CA, USA). The protocol followed was: 1X SsoAdvanced Universal SYBRGreen Supermix, 0.4 µM of FWD, 0.4 µM of REV, 50 ng of the template and a certain volume of water to obtain a final volume of 20 µL. The amplification was carried out by the instrument CFX96 Touch Real-Time PCR (Bio-Rad) using the same thermal cycle for all primer pairs, steadily changing the annealing temperature specifically for individual genes: initial denaturation at 95 °C for 1 min, followed by 44 cycles of denaturation at 95 °C for 30 s and annealing temperature for each specific gene for 30 s. The fluorescence was recorded at the end of each phase of polymerization. Each reaction was carried out in triplicate. The Melt Curve analysis was set up in a range from 60 °C to 95 °C, with temperature increments of 0.01 °C/s. The Ct values (threshold cycle) obtained from CFX Manager™ Software (Bio-Rad) showed a standard deviation lower than 0.2 the reactions performed in triplicate. The results were analyzed by GenEx-Pro, which is a software package designed for qPCR data processing and analysis (http://www.multid.se/index.html). The statistical significance was identified by analysis of variance (ANOVA) with R software (https://www.r-project.org/).

## 3. Results

### 3.1. PDGFA Full-Length Transcript

**Figure 1:**
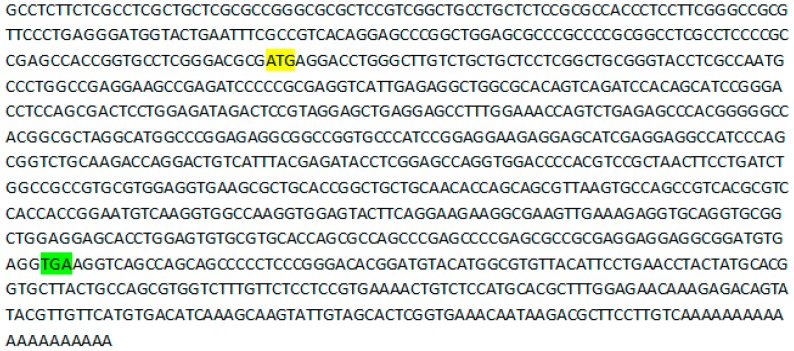
PDGFA full-length transcript. The sequence was taken from accession number MK026736. The start codon is highlighted in yellow. The stop codon is highlighted in green.

The characterization of the full-length cDNA of goat PDGFA revealed an open reading frame with a length of 591 bp, a 3′ UTR of 239 bp and a 5′ UTR of 182 bp, respectively. The 3′ UTR has a typical polyadenylation signal (AATAAA), followed by an additional 20-bp poly (A) tail (Figure 1).

The coding region obtained from the PDGFA cDNA sequence encodes a putative protein containing 196 amino acids (aa). The aa sequence was aligned with other PDGFA proteins and showed high similarity to Ovis aries (99%), Sus scrofa (98%), Canis lupus familiaris (98%), Homo Sapiens (98%), Rattus norvegicus (97%) (NCBI).

**Figure 2:**
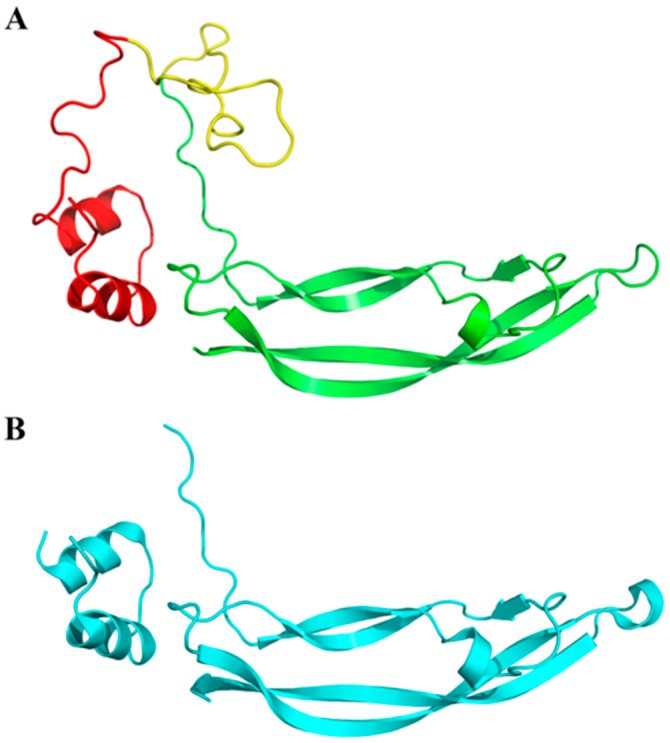
Goat PDGF subunit A3D protein structure. (**A**) The yellow structure is the signal peptide, red alpha-helix constitutes the propetide that is removed when the protein becomes mature; the green structures show the functional part, which are the beta sheets; (**B**) The human PDGF subunit Acrystallographic structure is reported for comparison.

The predicted protein structure is composed of different components: a signal peptide of 20 aa, a transient extension of the amino terminus of the protein, a propeptide with a length of 65 aa, which is removed when the protein becomes mature, and a mature protein with a length of 109. Through the Pymol program, which is used for 3D visualization of the proteins, we observed the very high structural identity of our protein with human PDGF subunit A based on the RMSD value (0.12 Å) (Figure 2).

### 3.2. Morphological Evaluation

**Figure 3:**
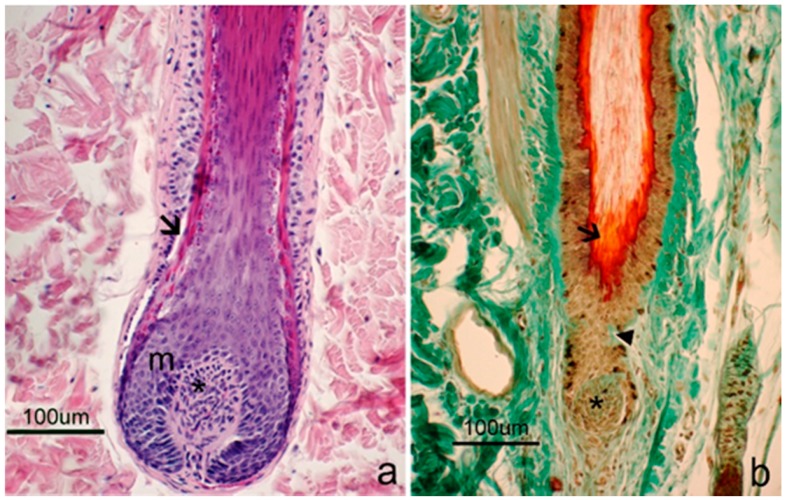
Hair follicles (HF) during different periods of the hair cycle. (**a**) The deepest part of an anagen HF is showed, which contains a large and onion-shaped bulb that extends into the dermis. The dermal papilla (*) consists of fibroblasts embedded in abundant extracellular matrix and small blood vessels. The dermal papilla is completely enclosed by matrix cells (m). The inner root sheath (arrow) is developed and clearly visible. Hematoxylin & Eosin staining; (**b**) A HF at the end of the regressive phase. The typical morphological features of this period are shown, which include a little and rounded dermal papilla (*); a shrunk matrix; a short epithelial strand (arrowhead); and a trichilemmal sac surrounding the hair shaft (arrow). Floxin B/Orange G/Alcian blue staining.

Goat skin samples were accurately analyzed to determine which stage of the follicular cycle they were in. HFs observed in the samples collected during the early anagen and anagen periods showed morphological characteristics that were typical of the growing phase, including deep and large bulbs (Figure 3a). In early catagen samples, most HFs still exhibited anagen characteristics although some follicles in the regressive phase, showing the epithelial strand and trichilemmal keratinization, were observed. In the late catagen phase, most secondary HFs confirmed this phase. They appeared short, without the bulb region and with their proximal part near to the attachment point of the arrector pili muscle. The inner root sheath was replaced by the trichilemmal keratinization (Figure 3b).

### 3.3. Immunohistochemical Evaluation

Anti-PDGFA and anti-PDGFRα antibodies reacted consistently with all tested skin samples and showed the expression of the molecules in some structures of goat skin, including the epidermis and HFs.

**Figure 4:**
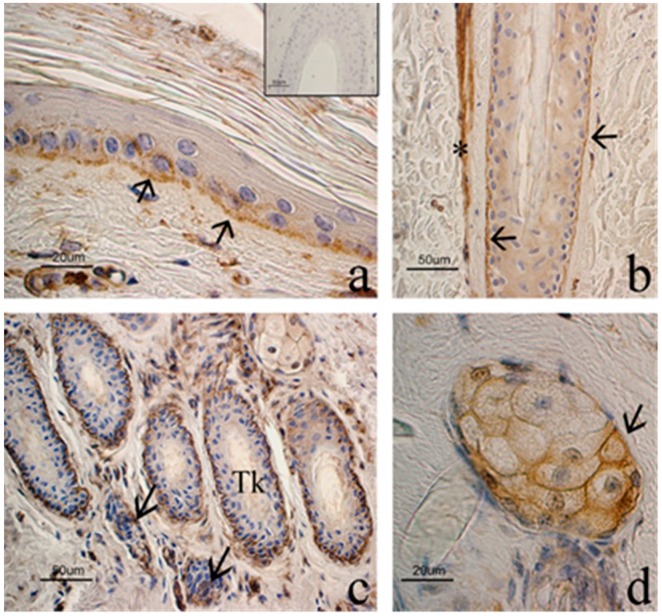
PDGFA expression in goat skin. (**a**) In the epidermis, the cells of the basal layer show a clear staining of the cell membrane (arrows), while suprabasal layers are negative. Negative control in the inset. (**b**) Isthmic region of an anagen hair follicle (HF): immunostaining is localized in the basal cells of the outer root sheath (arrows). * = arrector pili muscle. (**c**) A group of secondary regressive phase HFs, which are characterized by irregular edges of the outer root sheath and trichilemmal keratinization (TK). Basal cells surrounding the club hair show a strong immunostaining. Arrows point out two dermal papillae. (**d**) PDGFA expression in the sweat gland (arrow). ABC immunohistochemical staining. Nuclei are counterstained with Hematoxylin.

PDGFA was localized in the cells of the basal layer of the epithelium: the signal was clearly observed in the cell membrane and mainly involved the basal compartment (Figure 4). With regards to HF, immunostaining extended from the infundibulum to the proximal end of the isthmus while no staining was observed in the bulb. Staining involved the basal layer of the outer root sheath while the inner root sheath appeared to be inconsistently positive. PDGFA positivity was sharp in all stages of hair cycle although the regressive HFs showed a more intense signal that strongly demarcated the isthmic region surrounding the club hair of catagen and telogen HFs. During these phases of the hair cycle, the dermal papilla also showed positivity for PDGFA. Both primary and secondary HFs showed the same immunostaining pattern for PDGFA (Figure 4).

PDGFA was inconsistently observed in some basal cells of the sebaceous glands while sweat glands appeared to be negative. In all samples, smooth muscle cells showed an intense immunoreaction to PDGFA. Accordingly, the arrector pili muscle, myoepithelial cells of the sweat glands and tunica media of blood vessels were used as internal positive controls (Figure 4).

**Figure 5:**
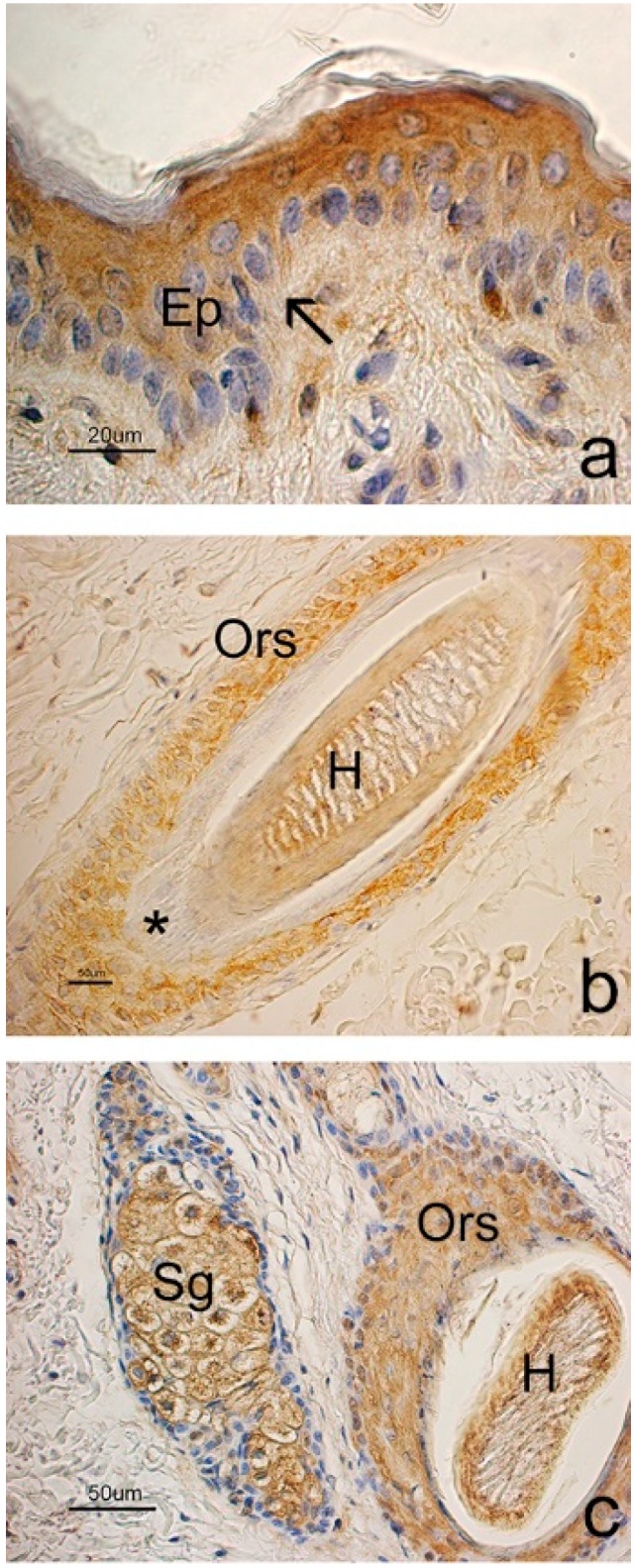
PDGFRα expression in the goat skin. (**a**) In the epidermis (Ep), immunostaining mainly extended in the suprabasal cell layers. Arrow points to basal cells. (**b**) Isthmic region of an anagen hair follicle (HF): immunostaining clearly involved all the cells of the outer root sheath (Ors) while inner root sheath was negative (*). A hair (H) is in the follicular canal. (**c**) A sebaceous gland (Sg): staining is mainly localized in the secretory cells while the gland basal cells are negative. On the right, a primary HF in a transverse section shows an intense staining to PDGFRα in the outer root sheath (Ors) cells. H = hair. ABC immunohistochemical staining. Nuclei are counterstained with Hematoxylin.

The receptor of PDGFA was observed in the epidermis and HFs (Figure 5). However, it was mainly expressed by the suprabasal cells and staining was clearly localized in the cell cytoplasm. The staining extended along the follicular wall from the infundibulum to the isthmus and involved the outer root sheath while the inner root sheath appeared to be negative. The receptor was observed in both primary and secondary HFs throughout the follicular cycle. PDGFRα was also observed in the secretory cells of the sebaceous glands.

### 3.4. qPCR Evaluation

**Figure 6:**
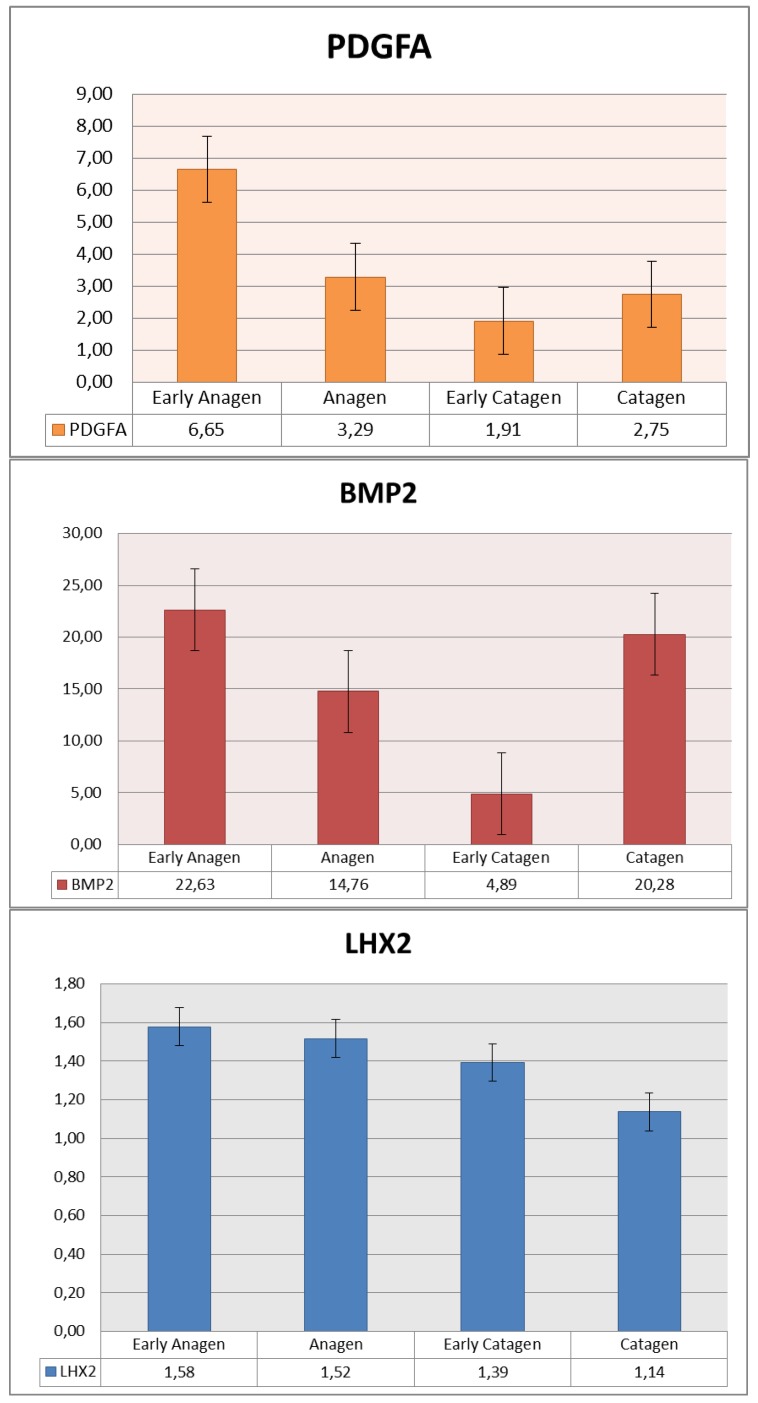
Expression levels of PDGFA, BMP2 and LHX2 during different phases of hair follicle (HF) cycle.

The expression levels of PDGFA, BMP2 and LHX2 were analyzed in four different times (June, September, December and January) on all cashmere goat skin samples collected (Figure 6). The qPCR was performed using three reference genes, which were namely SDHA, YWHAZ and UBC. Follicular cycle-related expression differences were observed for PDGFA and BMP2. PDGFA showed a peak during the early anagen phase, a decrease in the anagen and early catagen phases and finally a slight increase in the late catagen phase. The BMP2 expression levels showed a trend, which was similar to that of PDGFA. They were high during the early anagen and late catagen phases while they decreased during the anagen and early catagen phases. LHX2 expression levels were quite similar in all four phases although a slight and progressive decreasing trend from the early anagen to late catagen phase was observed. The ANOVA test (*p* < 0.005) was performed to analyze the results (Table 3).

This showed that LHX2 had no significant variations in expression levels during the different HF cycle phases while significant variations in levels of expression were observed for PDGFA and BMP2 (Figure 6).

## 4. Discussion

Similar to other mammals, such as sheep and mink, many breeds of goat have a double coat composed of the guard hairs produced by primary HFs and the fine down (cashmere) underwool hairs produced by the secondary HFs [33]. The coat experiences photoperiodic-dictated alterations, which prepares the animal for changes in ambient temperature. This is driven by HFs, which undergo regular cycles of involution and regeneration. It is unquestionable that the generation of a new hair depends on the activation of hair-specific epithelial stem cells, which are located in the bulge region of the HF that serves as a reservoir for epithelial and sebaceous gland cells [7]. The activation of HF stem cells is driven by members of several families of signaling molecules.

PDGFA, which is secreted by the adipocyte precursor cells, was suggested to be instrumental in HF regeneration during the hair cycle [11,14]. In this work, PDGFA was studied to evaluate its role in goat HF by analyzing its expression in the skin of selected young female cashmere goats throughout the HF cycle. Using skin biopsies, PDGFA mRNA was sequenced for the first time in goats. The comparison of the genes, which was performed using GenBank sequences, revealed that goat PDGFA gene is similar to Ovis aries (99%), Sus scrofa (98%), Canis lupus familiaris (98%), Homo Sapiens (98%) and Rattus norvegicus (97%) (NCBI). This high homology suggests a common function or mechanism of this molecule across species. Cells expressing PDGFA and its receptor in cashmere skin were identified by immunohistochemistry to identify the skin structures that produce and are reactive to PDGFA. In all samples, HFs showed the expression of both molecules. In particular, PDGFA immunostaining was observed in the outer root sheath cells from the infundibulum to the proximal end of the isthmus. The expression of PDGFA was already described in the follicular epithelium of the upper part and the bulge region in mouse and human fetuses [34,35]. Accordingly, it was hypothesized that PDGFA plays a role in HF morphogenesis [36]. The PDGFA receptor was observed in the follicular epithelium, suggesting that HF is a responsive structure to the actions of PDGFA according to Kamp et al. [37] who described the expression of the receptor in human follicular keratinocytes. The localization of both PDGFA and PDGFRα in the outer root sheath cells suggests that PDGFA acts on HF by an autocrine and paracrine mechanism [37]. Furthermore, this supports HF having a primary role in PDGFA secretion and in the regulation of its own cycle. PDGFA persisted throughout follicular cycle although there was stronger immunostaining in the cells of the isthmic region surrounding the club hair. PDGFA qPCR evaluation confirmed immunohistochemical data. The molecular expression was high in the late catagen and early anagen phase, which occurs during the passage between two follicular cycles when the HF starts to prepare the new cycle. The stronger expression of PDGFA observed in this period confirms that this molecule is involved in HF growth and likely activates the anagen phase as previously hypothesized in other species [12,17].

Together with PDGFA, the expression of BMP2 and LHX2 was analyzed by qPCR throughout the cashmere HF cycle since literature has suggested that they are anagen activators [13,18,19].

In this study, BMP2 behaved in a similar way to PDGFA with high levels in the early anagen phase, which were lower during the anagen and early catagen phase before increasing in the late catagen phase. This corroborates the findings of the study conducted by Su et al. [19], which suggested that BMP2 may play a role in the regeneration of HFs and is involved in the regulation of follicular morphogenesis.

LHX2 is described as an important regulator of HF cycle as it controls the switch between stem cell maintenance and activation in the HFs [23]. In the cashmere goat analyzed in this study, LHX2 showed a slight but progressive decrease in its expression levels from the early anagen to late catagen phase. Many authors connected this gene with the anagen phase when the new hair cycle begins [24]. Geng et al. [18] showed that LHX2 decreased from the anagen phase to other phases in cashmere goats and Wang et al. [25] confirmed that in goat LHX2 regulates the generation and regeneration of the hair. However, the differences observed in the present study were very faint and not significant, which suggests that LHX2 mainly maintains a continuous basal level throughout follicular cycle and cannot be primarily considered as an anagen activator in cashmere goats.

## 5. Conclusions

PDGF is a potent mitogen produced in a variety of cell types. It is important for cell growth, proliferation and differentiation as it induces and maintains the anagen phase in mouse HF. In this research, the expression of some molecular signals implicated in hair growth, including PDGFA, BMP2 and LHX2, was identified and the full-length transcript of goat PDGFA gene was sequenced. The goat hair cycle is influenced by the photoperiod, which shows rhythmic activity of the growth. Stem cells are involved in the cycle activation and start to differentiate in the early phase of the growth. In this study, PDGFA and BMP2 genes were confirmed as activators of HF stem cells while LHX2 was suggested to play a basic role during the hair growth with a continuous and non-fluctuating presence.

## Figures and Tables

**Table 1:** PDGFA gene primers.

Primer	Sequence	TM (°C)
FWDA	CTCGGGACGCGATGAGGAC	60.3
FWDB	GATGAGGACCTGGGCTTG	58.2
OLIGODT	GAGAGAGAGAGAGACAGAGAACTAGTCTCGAGTTTTTTTTTTTTTTTTTT	74.9

**Table 2:** Primer designed for Real-Time PCR with Efficiency.

Primer	Sequence (Forward and Reverse)	TM (°C)	Efficiency (100%)
*PDGFA*-	TCCGCTAACTTCCTGATCT CTTTCAACTTCGCCTTCTT	56	95.6
*BMP2*-	CTACATGCTGGACTTGTAC GTTGTTTTCCCACTCATTTC	55	83.3
*LHX2*-	GGAAGCATCTACTGCAAGGAAG GAGGTGATAAACCAAGTCCCG	55	93.2
*UBC*	GCATTGTTGGGTTCCTGTGT TTTGCATTTTGACCTGTGAG	57	116.2
*YWHAZ*	TGTAGGAGCCCGTAGGTCATCT TTCTCTCTGTATTCTCGAGCCATCT	57	105
*SDHA*	AGCACTGGAGGAAGCACAC CACAGTCGGTCTCGTTCAA	57	128.2

**Table 3:** Analysis of variance (ANOVA).

Gene	*p* Value
*PDGFA*	8 × 10^−8^
*BMP2*	0.00024102
*LHX2*	0.04075542
